# Application of Artificial Intelligence to Advance Individualized Diagnosis and Treatment in Emergency and Critical Care Medicine

**DOI:** 10.3390/diagnostics14070687

**Published:** 2024-03-25

**Authors:** Jie Yang, Bo Zhang, Xiaocong Jiang, Jiajie Huang, Yucai Hong, Hongying Ni, Zhongheng Zhang

**Affiliations:** 1 Department of Emergency Medicine, Sir Run Run Shaw Hospital, Zhejiang University School of Medicine 3#, East Qingchun Road, Hangzhou 310016, China; 22218206@zju.edu.cn (J.Y.); 3414285@zju.edu.cn (B.Z.); jxc506180@163.com (X.J.); 22218207@zju.edu.cn (J.H.); realhealth@zju.edu.cn (Y.H.); 2 Department of Critical Care Medicine, Affiliated Jinhua Hospital, Zhejiang University School of Medicine, No.365 Renmin East Rd, Jinhua 321000, China

Emergency and critical illnesses refer to severe diseases or conditions characterized by rapid changes in health that may endanger life within a short period [1]. With the development of information technology [2], the storage, sharing, and use of medical data have become more convenient. Furthermore, with the rapid development of artificial intelligence technology [3,4], we are at the forefront of a medical information revolution. The emergence of AI has brought new possibilities for the treatment of critically ill patients [5,6]. Through big data analysis and machine learning, we are able to predict the progression of diseases more accurately and adjust treatment plans in a timely manner [7,8,9]. This trend towards precision medicine has had a profound impact on medical practice [10]. In this context, we have organized a Special Issue to discuss the application of AI in the management of critical illnesses, aiming to achieve greater advancements in future healthcare.

In this Special Issue, researchers from various countries and regions have explored the application of artificial intelligence in critical care, covering aspects such as diagnosis, management, and prognosis. (Figure 1) Several previous studies have explored the realm of diagnosis. Aygun et al. (Contribution 1) aimed to develop an interpretable predictive model based on Explainable Artificial Intelligence (XAI) to forecast sepsis and identify significant biomarkers. Within a cohort of 1572 patients, they utilized biomarkers including age, respiratory rate, blood oxygen saturation, procalcitonin, and positive blood culture to predict sepsis. The results of SHapley Additive exPlanations (SHAP) indicate that factors such as advanced age, increased respiratory rate, and decreased procalcitonin elevate the risk of sepsis. By enhancing transparency in the decision-making process, XAI models enable clinicians to comprehend and trust the predictive capabilities of AI systems [11]. During the diagnostic process, multiple methods are often available to assist physicians in making a diagnosis. Tuncyurek (Contribution 2) conducted an evaluation using artificial intelligence methods to determine the optimal selection of methods. Acute appendicitis stands as one of the most common causes of abdominal pain in the emergency department and is the leading surgical emergency among children under 15, posing a significant risk upon rupture. The selection of radiological methods is paramount for accurate diagnosis, thereby averting unnecessary surgeries. The aim of this study is to evaluate the effectiveness of the American College of Radiology (ACR) Appropriateness Criteria in diagnosing acute appendicitis using multivariable decision criteria. This study’s uniqueness lies in its provision of an analytical ranking of results for this intricate decision problem, showcasing the merits and demerits of each alternative in different scenarios, even accounting for the ambiguity of emergency situations concerning the diagnosis of pediatric appendicitis. We applaud the utilization of a novel model to test the ACR qualification criteria, aiming to minimize confusion in the diagnostic process.

Figure 1 provides a visual summary of the contents covered in the Special Issue. Through these visual elements, readers can quickly grasp the various topics and any related content featured in the Issue. XAI: Explainable Artificial Intelligence; TBI: Traumatic Brain Injury; ICU: Intensive Care Unit; AKI: Acute Kidney Injury; COVID-19: Coronavirus Disease 2019; VAP: Ventilator-Associated Pneumonia; NPA: Neonatal Pain Assessment; OS-NPA: On-Site Neonatal Pain Assessment; ACR: The American College of Radiology.

The risk of acute kidney injury (AKI) has long presented a challenge for clinicians in the intensive care unit (ICU) [12,13]. The combined effects of serum creatinine, blood urea nitrogen (BUN), and clinically relevant serum electrolytes have yet to be comprehensively studied. Through a screening of the MIMIC-IV Database, the association between serum electrolyte levels and renal function was examined (Contribution 3), revealing that levels of serum creatinine, chloride, and magnesium emerged as the three primary factors requiring monitoring in this patient cohort. Thus, it is imperative to undertake larger-scale studies based on this research to strengthen and refine the clinical guidelines pertaining to AKI.

Rambaud et al. developed a clinical prediction algorithm using prospective clinical data from 827 pediatric patients stored in a Canadian tertiary pediatric hospital database (Contribution 4). This algorithm aims to enable the early detection of ventilator-associated pneumonia (VAP). Notably, this study demonstrates the most accurate sensitivity achieved by a Clinical Decision Support System (CDSS) to date in identifying VAP. We anticipate the implementation of the results of this algorithm by Jerome Rambaud in the Pediatric Intensive Care Unit (PICU), and we look forward to seeing its outstanding performance in multicenter trials.

Furthermore, artificial intelligence has numerous applications in prognosis. In the fields of traumatic brain injury (TBI) and burn injuries, artificial intelligence (AI) technologies have significantly advanced the predictive accuracy of patient outcomes beyond traditional models. Tu et al. and Yeh et al. conducted retrospective studies in their respective areas, gathering extensive patient data to employ machine learning models in the prediction of mortality and adverse outcomes. The researchers focused on 2260 TBI patients in the ICU, using four machine learning models and 42 features. Their study outperformed traditional tools like APACHE II and SOFA scores in predicting mortality risk (Contribution 5). Similarly, Yeh et al. analyzed data from 348 burn patients, demonstrating AI’s ability to predict prolonged hospital stays, grafting needs, and other adverse outcomes more accurately than the commonly used Baux score (Contribution 6). Both studies highlight the role of modern AI in enhancing the stratification and assessment of patients, marking significant progress towards intelligent healthcare in their respective domains. In another study (Contribution 7) utilizing the MIMIC-IV Database, multiple machine learning models were employed to predict patient mortality, with the findings indicating that the XGBoost machine learning method outperformed traditional models through comparative analysis.

Furthermore, artificial intelligence plays a significant role in disease management [14]. Amidst the outbreak of the COVID-19 pandemic, the escalating number of critically ill patients in global intensive care units (ICUs) has placed a strain on ICU resources. The early prediction of ICU demand is crucial for effective resource management and allocation. Islam et al. conducted a retrospective cohort study focusing on data collected from the Pulmonology Department of a state hospital in Moscow (Contribution 8). Various feature selection techniques were investigated, and a stacked machine learning model was proposed. This model was compared with eight different classification algorithms to assess the risk of ICU admission for both COVID-19 and non-COVID patients, as well as for COVID patients separately.

Artificial intelligence also plays an important role in image recognition and assessment in the medical fields. The assessment of neonatal pain (NPA) has not received sufficient attention in clinical practice, leading to widespread instances of undertreatment of pain severity. In clinical NPA, facial expressions are considered the most explicit indicators, upon which various pain assessment scales are designed. Zhu et al. aimed to develop an automated NPA system that meets actual clinical needs (Contributions 9 and 10). To achieve this, a video database capturing the facial expressions of neonates during blood collection procedures in the neonatal ward was established, and an AI-NPA method was developed based on real-world data. The clinical utility of the automated NPA system was validated by recruiting 232 pediatric patients from a tertiary children’s hospital in China. According to the OS-NPA results of 232 neonates, the accuracy of the automated NPA system was 88.79%. Although video-based NPA (VB-NPA) protocols facilitate remote or post hoc pain diagnosis by experts, serving as an equivalent alternative to the on-site NPA (OS-NPA) gold standard, VB-NPA may suffer from partial inaccuracies due to information loss in neonatal pain videos captured in real NICU settings. Nonetheless, it remains comparable to OS-NPA. Video-based assessment for neonatal pain evaluation in clinical settings is feasible and allows for the real-time, remote assessment of neonatal pain severity. We anticipate further applications of this technology in neonates with larger-scale data and potential migration to adult pain assessment.

Collectively, these studies illuminate the transformative impact of AI and ML in medical diagnostics and prognostics, heralding a new era of precision medicine that promises enhanced patient outcomes and optimized healthcare delivery [15]. Additionally, in the application of artificial intelligence, there are characteristics of overfitting and poor generalization; some models produce very precise predictions, but this precision may not necessarily translate into clinical benefits [16]. To address these issues of generalization, further validation is needed through randomized controlled trials (RCTs) across multiple centers.

## Figures and Tables

**Figure 1:**
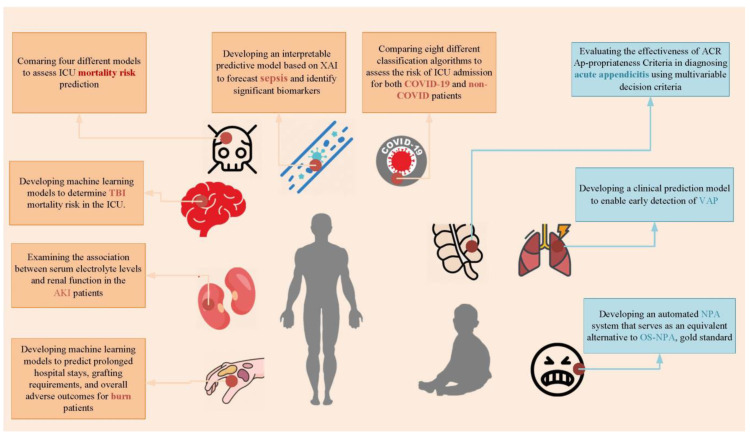
The figure provides a visual summary of the contents covered in the special issue. Through these visual elements, readers can quickly grasp the various topics and related content featured in the issue. XAI: Explainable Artificial Intelligence; TBI: Traumatic Brain Injury; ICU: Intensive Care Unit; AKI: Acute Kidney Injury; COVID-19: Coronavirus Disease 2019; VAP: Ventilator-Associated Pneumonia; NPA: Neonatal Pain Assessment; OS-NPA: on-site Neonatal Pain Assessment; ACR: the American College of Radiology.
